# Evaluation of veteran community care outcomes after coronary artery bypass grafting: a retrospective pilot cohort

**DOI:** 10.1186/s13019-024-02644-8

**Published:** 2024-03-26

**Authors:** Jake L Cotton, Adom Netsanet, Alejandro Suarez-Pierre, Danielle Abbitt, Teresa S Jones, Jessica Y Rove, Edward L Jones

**Affiliations:** 1 Department of Surgery, University of Colorado School of Medicine, 12631 East 17th Avenue, 6111, 80045 Room, Aurora, CO USA; 2 Department of Surgery, Rocky Mountain Regional VA Medical Center, Aurora, CO USA; 3 Division of Cardiothoracic Surgery, Department of Surgery, University of Colorado School of Medicine, Aurora, CO USA

**Keywords:** Veterans health, Patient outcome assessment, Coronary artery bypass, VHA

## Abstract

For Veterans who cannot be seen in a timely fashion or must travel long distances to be seen, the Veterans Health Administration (VHA) offers funded care in the community. The use of this program has rapidly increased; however, there have been no systematic evaluations of surgery specific metrics such as perioperative complications, mortality and timeliness of care. To evaluate this in cardiac surgery patients, we compared veterans undergoing coronary artery bypass grafting in the community to those remaining within the VHA. We identified 78 patients during calendar year 2018 meeting inclusion criteria. 41 underwent surgery in the community versus 37 in the VHA. There were no significant differences in baseline demographics including age, sex, race, ethnicity, comorbidities and surgical risk scores. With regard to perioperative outcomes, veterans who underwent surgery within the VHA had lower infection rates (17% vs. 0%, *p* = 0.008) and 30-day emergency department utilization (22% vs. 5%, *p* = 0.04). A longer median postoperative inpatient stay was also seen within the VHA (8 days vs. 6 days, *p* < 0.001). These findings suggest that the VHA may better serve Veterans and prevent adverse events after CABG, at the expense of prolonged hospitalization. More study is needed to validate the findings of this pilot study.

## Introduction

Since 2014, the Veterans Healthcare Administration (VHA) has provided a funded community care option when patients are unable to be seen in a timely fashion or must travel long distances. In fiscal year 2020, 2.3 million Veterans were enrolled in community care with an expenditure of $16.9 billion [1]. Projections estimate a 25% expenditure increase over the following three years. Despite increasing enrollment, there is lack of data to support the quality of community care for cardiac surgery [1]. Prior studies have demonstrated that when compared with private institutions, the VHA provides equivalent or superior surgical care using general metrics including 30 and 90-day mortality, emergency department (ED) visits, and readmission [2, 3]. A comprehensive review of community care should evaluate surgery specific outcomes [4]. We sought to evaluate surgery specific outcomes after community care coronary artery bypass grafting (CABG) compared with the VHA. We hypothesized that superior surgery specific outcomes would be found within the VHA.

## Methods

A retrospective, pilot study was designed and identified Veterans undergoing isolated CABGs within the Rocky Mountain Regional Veterans Affairs Medical Center (RMR VAMC) referral area between January 1st, 2018, and December 31st, 2018. Veterans were identified via the corporate data warehouse (CDW), a central VHA administrative database, using CABG current procedural terminology codes (33,510–33,514, 33,516–33,519, 33,521–33,523, 33,530, and 33,533–33,536). Demographics including age, sex, race, ethnicity, vital status, and date of death were obtained from the CDW and perioperative variables were collected through manual chart review for VHA-performed CABGs and scanned record review in Vista Imaging made available by community care facilities for all VHA-paid care. Records reviewed included pre-operative history and physicals, operative reports, hospital progress notes, and discharge summaries, which confirmed isolated CABG via conventional sternotomy. Isolated CABG was defined as no simultaneous aortic or valve repair or replacement. Patients with ED visits or readmissions within 30 days also had those records reviewed. Records without a preoperative history and physical, operative report and/or discharge summary were excluded from the study to minimize information bias. Non-parametric continuous variables were compared using the Wilcoxon rank sum test and categorical variables were compared using the chi-square test (*n* > 5) or Fisher’s exact test (*n* ≤ 5). Statistical significance was defined as *P* < 0.05. Analyses were performed using R version 4.2.1 (The R Foundation, Vienna, Austria). This study was approved by the Colorado Multiple Institutional Review Board (COMIRB #19-2384).

## Results

Seventy-eight patients met the inclusion criteria, 41 undergoing CABG in the community and 37 within the VHA. Thirteen patients (13/54 [24%]) were excluded from the community care cohort due to incomplete records. There was no difference in baseline characteristics, Table 1.

**Table 1:** Baseline Characteristics

	VHA Care (*n* = 37)	Community Care (*n* = 41)	*P* value
Age (years)	67 [63–70]	68 [61–72]	0.81
Male Sex	37/37 (100%)	40/41 (98%)	> 0.99
Race			
American Indian/Alaska Native	0/37 (0%)	2/41 (5%)	0.05
Black	3/37 (8%)	0/41 (0%)	
White	34/37 (92%)	39/41 (95%)	
Ethnicity			
Hispanic/Latino	1/37 (3%)	4/41 (10%)	0.50
Not Hispanic/Latino	34/37 (92%)	35/41 (85%)	
Unknown	2/37 (5%)	2/41 (5%)	
Tobacco Use			
Never	9/37 (24%)	9/41 (22%)	0.93
Current	20/37 (54%)	21/41 (51%)	
Former	7/37 (19%)	10/41 (24%)	
Unknown	1/37 (3%)	1/41 (2%)	
BMI (kg/m^2^)	31.2 [27.9–34.1]	30.5 [27.3–35.9]	0.74
Prior Cardiac Surgery	0/37 (0%)	1/41 (3%)	> 0.99
Atrial Fibrillation	6/37 (16%)	3/41 (7%)	0.30
Prior CVA	3/37 (8%)	3/41 (7%)	> 0.99
COPD	7/37 (19%)	12/41 (29%)	0.42
Diabetes	19/37 (51%)	21/41 (51%)	> 0.99
Peripheral Vascular Disease	3/37 (8%)	5/41 (12%)	0.44
CKD	13/37 (35%)	13/41 (32%)	0.94
EF (%)	55.0 [52.5–60.0]	52.0 [39.4–60.0]	0.10
VASQIP 30-Day Mortality (%)	0.86 [0.58–1.30]	0.79 [0.54–1.59]	0.81
STS Mortality Risk (%)	0.89 [0.67–1.65]	1.27 [0.82–2.11]	0.08

VHA = Veterans Health Administration; BMI = Body Mass Index; CVA = Cerebrovascular Accident; COPD = Chronic Obstructive Pulmonary Disease; CKD = Chronic Kidney Disease; EF = Ejection Fraction; VASQIP = Veteran Affairs Surgical Quality Improvement Program; STS = Society of Thoracic Surgeons

Continuous variables reported as median [IQR]; Categorical variables reported as number (percent)

Most CABGs were urgent (VHA: 25/37 [68%] and community: 30/41 [73%]). Veterans undergoing CABG within the VHA had lower infection rates and 30-day ED utilization, although longer postoperative stays, Table 2. Postoperative infections after community care CABGs included surgical site infections (4/41 [10%]), pneumonia (2/41 [5%]), and urinary tract infections (1/41 [2%]). The reasons for ED utilization after community care CABGs were heart failure (3/41 [7%]), arrhythmias (2/41 [5%]), surgical site infections (1/41 [2%]), chest pain (1/41 [2%]), critical limb ischemia (1/41 [2%]), and hypotension (1/41 [2%]). The reasons for ED utilization after VHA CABGs were chest pain (1/37 [3%]) and cough (1/37 [3%]).

**Table 2:** Postoperative quality metrics

	VHA Care (*n* = 37)	Community Care (*n* = 41)	*P* value
Vessels Bypassed	3 [2-3]	3 [3-4]	< 0.001*
Cardiopulmonary Bypass Time (minutes)	121 [88–145]	93 [72–149]	0.19
Cross-Clamp Time (minutes)	81 [63–100]	67 [45–92]	0.13
Atrial Fibrillation	14/37 (38%)	17/41 (41%)	0.74
Prolonged Intubation^a^	6/37 (16%)	6/41 (15%)	0.85
Infections	0/37 (0%)	7/41 (17%)	0.008*
Length of Stay (days)	8 [6–14]	6 [3–7]	< 0.001*
30-Day ED Utilization	2/37 (5%)	9/41 (22%)	0.04*
30-Day Readmission	3/37 (8%)	3/41 (7%)	0.90
30-Day Mortality	1/37 (3%)	1/41 (2%)	> 0.99

VHA = Veterans Health Administration; ED = Emergency Department

^a^Prolonged intubation defined as > 24 h post-operatively

Continuous variables reported as median [IQR]; Categorical variables reported as number (percent)

*P < 0.05

## Discussion

This pilot study is the first to evaluate VHA-supported cardiac surgical care, both in the community and at VHA facilities. The cohorts were similar in all evaluated baseline characteristics despite prior studies identifying community care patients more often being female, younger, and with fewer comorbidities [2]. The lower infection rate for patients undergoing surgery in the VA may reflect practice differences such as prophylactic antibiotic duration, Foley duration, and adherence to a pulmonary hygiene regimen. Care coordination, rapid access clinics available through the VHA, and early contact after discharge may account for the lower rate of ED utilization after VHA CABGs [5]. This is also reflected by the similar readmission rates despite differences in ED utilization, demonstrating the emergency care does not seem to necessitate readmission. The longer postoperative length of stay within the VHA may be attributed to the use of step-down units in the community, which is not available at the RMR VAMC, and are known to decrease post-intensive care length of stay [6]. These pilot findings suggest VHA care may better serve Veterans and prevent adverse events after CABG. Strengths of this pilot study include methodology that allowed for chart review of included records, minimizing inaccuracies from database inquiries. These methods can serve as a standard for validating quality of community care as the Veterans Affairs Surgical Quality Improvement Program has done for the VHA and the National Surgical Quality Improvement Program has done for enrolled facilities. The authors recognize limitations of this pilot cohort and conclusions should be interpreted within the context of its limitations. Limitations of our study include intrinsic limitations of an unmatched retrospective cohort review, possibility of missing records from community care hospitals, and a small sample size. As an unmatched retrospective cohort review, it is not possible to control for every variable including unevaluated baseline characteristics and differences in community care hospital resources. There is a chance of a difference in baseline severity of coronary artery disease as reflected in the difference in number of vessels bypassed. Alternatively, the influence of increasing reimbursement for additional anastomoses has not been studied and warrants further investigation. Risk of bias from missing records was minimized by only including patients with history and physicals, operative reports, and discharge summaries available for review. Furthermore, it is possible records from subsequent community care encounters (ED utilization and readmission) are missing from patient charts, although this risk exists for both veterans undergoing CABG within the VHA and the community. The small sample size increases risks of type II errors in preoperative characteristics and prevents meaningful evaluation of wait time differences. Future work will include a larger cohort and comparison of surgical wait times and cost differences.

## Data Availability

The datasets generated and analyzed during the current study are not publicly available due to possibility of identifying information contained within.

## Abbreviations

VHA: Veterans Health Administration
ED: Emergency Department
CABG: Coronary Artery Bypass Grafting
RMR VAMC: Rocky Mountain Regional Veterans Affairs Medical Center
CDW: Corporate Data Warehouse
BMI: Body Mass Index
CVA: Cerebrovascular accident
COPD: Chronic Obstructive Pulmonary Disease
CKD: Chronic Kidney Disease
EF: Ejection Fraction
VASQIP: Veterans Affairs Surgical Quality Improvement Program
STS: Society of Thoracic Surgeons
